# Research on Bone Cells in Health and Disease

**DOI:** 10.3390/ijms25168758

**Published:** 2024-08-12

**Authors:** Dávid S. Győri

**Affiliations:** Department of Physiology, Semmelweis University School of Medicine, 1094 Budapest, Hungary; gyori.david@semmelweis.hu; Tel.: +36-1-459-1500 (ext. 60345)

Bone-forming osteoblasts, osteocytes, and bone-resorbing osteoclasts are responsible for life-long skeletal remodeling. Their abnormal function contributes to the development of bone diseases such as osteoporosis, osteopetrosis, tumor-induced osteolysis, and inflammatory arthritis-related bone loss [1]. This Special Issue entitled “Research on Bone Cells in Health and Disease” in the *International Journal of Molecular Sciences* provides new insights into the cellular and molecular mechanisms underlying bone formation and resorption, with a special focus on the activation of osteoblasts, osteocytes, and osteoclasts in physiological and pathological conditions.

Osteoporosis is a systemic skeletal disease characterized by low bone mineral density and microarchitectural deterioration of bone tissue that results in fragility fractures. In Western societies and developed countries with an aging population, the incidence of senile, postmenopausal, and glucocorticoid-induced osteoporosis increases dramatically [2]. Osteopetrosis is characterized by enhanced bone density, abnormal bone formation, and highly mineralized cartilage. It is also called “marble bone disease”, where enhanced bone density is considered to be a consequence of failure in bone resorption [3]. Osteolytic bone metastases, where bone tissue is destroyed, lead to pathological fractures and increased patient mortality [4]. Although cancer cells forming skeletal lesions are able to exert proteolytic activity to some degree, they cannot break down the bone matrix. Evidence suggests that interaction between tumor and bone cells is essential for successful osteotropic tumor cell metastatic dissemination. Finally, inflammatory arthritis is a manifestation of chronic inflammation affecting the synovial membrane of the joints and the periarticular bone. Bone destruction in inflammatory arthritis is best described in rheumatoid arthritis, where inflammation of the synovial joints is accompanied by cartilage and bone destruction [5].

Osteoblasts are involved in the formation of bone tissue and in skeletal remodeling. They are derived from mesenchymal stem cells via the activation of the Wnt/β-Catenin signaling pathway. Their precursors are recruited to bone by growth factors such as TGF-β and IGF-14, which are released from the bone matrix during bone resorption [6]. Mature osteoblasts are found to be present on the surface of the newly formed bone in a monolayer. They secrete the osteoid matrix at the edges of old bone tissue, where new bone formation takes place. The osteoid is the organic part of the extracellular matrix of the bone, and it is composed primarily of type I collagen. Due to the production of the osteoid matrix and the activation of the enzyme called alkaline phosphatase in the membrane of the osteoblast, the osteoid around the osteoblast begins to calcify, and hydroxyapatite crystals appear. Finally, mature osteoblasts transit to osteocytes or undergo apoptosis [6].

Osteocytes embedded in the bone matrix were considered to be able to regulate bone remodeling by activating both bone-forming osteoblasts and bone-resorbing osteoclasts. Recent evidence indicates that besides the above-mentioned important functions, osteocytes also have a higher capacity to induce osteoclastogenesis than other stromal cells of the bone, and they are the major source of growth factors and differentiation inducers for osteoclast development [7].

The other key cell type involved in the pathogenesis of the previously mentioned diseases is the osteoclast, the unique bone-resorbing cell of the human body [8]. Physiologically, osteoclast development is directed by two main growth factors, receptor activator of NF-κB ligand (RANKL) and macrophage colony-stimulating factor (M-CSF), which are provided by stromal cells such as osteoblasts [9]. The first phase of osteoclast development is then determined by the expression of various osteoclast-specific genes, such as tartrate-resistant acidic phosphatase (TRAP) and nuclear factor of activated T cells 1 (NFATc1) in pre-osteoclasts [10]. During the second phase of osteoclast differentiation, fusion of these pre-osteoclasts happens, leading to the development of large, multinucleated, mature osteoclasts [11]. Finally, those mature polykarions spread over the bone surface by forming actin rings and sealing zones in order to degrade the bone matrix via the parallel release of digestive enzymes and hydrochloric acid [12].

The first study in this Special Issue, by Mourão et al. [13], characterizes the effects of platelet-rich fibrin (PRF) on human primary osteoblasts (pOBs). Importantly, PRF promoted pOBs proliferation, independent of high (710 g) or low (44 g) relative centrifugal force (RCF)-based separation, while low RCF PRF contained higher quantities of TGF-β, leukocytes, and platelets. These findings may contribute to a better understanding of the cellular mechanisms of bone regeneration and can help to improve patient outcomes by advancing regenerative medicine.

The next study by Hasegawa et al. [14] examines minimodeling-based bone formation between the epiphyses and metaphyses of the long bones of eldecalcitol (ELD)-treated ovariectomized (OVX) rats. According to their results, ELD administration augmented bone volume and trabecular thickness by decreasing the number of osteoclasts in both the epiphyses and metaphyses of OVX rats. The epiphyses of the long bones of the ELD-treated animals demonstrated significantly increased minimodeling-based bone formation compared to remodeling-based bone formation. Further, the minimodeling-induced new bone formation contained few sclerostin-positive osteocytes, while the underlying pre-existing bone harbored many. Therefore, authors conclude that ELD can induce minimodeling-based bone formation in the epiphyses rather than in the metaphyses, and ELD-driven minimodeling may be associated with the inhibition of sclerostin synthesis.

The next research, conducted by Sung-Ju Lee and his colleagues [15], investigates the impact of the aqueous ethanol extract of Radix Asteris (EERA) on osteoclast differentiation and its contribution to osteoporosis management. They found that EERA retained osteoclast differentiation by inhibiting RANKL expression and RANKL-induced osteoclastogenesis. EERA strongly decreased RANKL-dependent expression of NFATc1, a key master regulator of osteoclastogenesis. When the authors conducted a phytochemical analysis of EERA, they identified several constituents involved in the regulation of bone and fat metabolism. Thus, their findings highlight the potential of EERA in the regulation of osteoclast development and may represent therapeutic potential for postmenopausal osteoporosis associated with metabolic imbalances.

The first review article in this Special Issue, by Chaekyun Kim [16], focuses on the roles of extracellular signal-regulated kinases (ERKs) in osteoclast differentiation. ERKs, p38, and c-Jun N-terminal kinases belong to mitogen-activated protein kinases (MAPKs) and play critical roles in regulating osteoclast proliferation, differentiation, and function. In most circumstances, ERKs positively regulate osteoclast development and function. However, inhibition of osteoclast differentiation by ERK1/2 was also observed, e.g., in RAW 264.7 cell-derived osteoclasts, by different research groups. The current understanding of the essential but contrasting roles of ERK1/2 during osteoclast development is summarized in this review.

Finally, in the last review article in this Special Issue, Sandor et al. [17] highlight the local effects of steroid hormones within the bone microenvironment and during the development of osteolytic bone metastases. Recently, accumulating evidence indicated the role of steroid hormones produced locally within the nervous and immune systems, skin, adipose tissue, and intestine [18,19]. This so-called extraglandular steroidogenesis is independently controlled from the feedback regulatory mechanisms of the hypothalamus–pituitary–steroidogenic gland axis regulating the hormone secretion of the adrenal cortex, gonads, and placenta (also known as glandular steroidogenesis). Bone cells, i.e., osteoblasts, osteocytes, and osteoclasts can respond to steroid hormones produced within the bone microenvironment, and our group recently identified a key role for extranglandular *de novo* steroidogenesis in osteotropic tumor cells during osteolytic skeletal lesion formation [20]. The identification of the steroid pregnenolone and its derivates, which play important roles in the regulation of bone homeostasis and the metastatic dissemination of cancer cells, is also discussed in detail in this review.

A better understanding of the molecular mechanism of osteoblast, osteocyte, and osteoclast activation in health and disease may facilitate the development of novel therapies for the treatment of skeletal diseases associated with pathological bone remodeling, e.g., osteoporosis, osteopetrosis, tumor-induced osteolysis, and inflammatory arthritis-related bone loss. For instance, current anti-resorptive therapies that block osteoclastogenesis by targeting RANKL (e.g., denosumab) or promote the apoptosis of osteoclasts (e.g., bisphosphonates) likely inhibit osteoclast-osteoblast and osteoclast-osteocyte interaction as well, since osteoclasts are known to release factors that regulate osteoblast and osteocyte activity [21]. Therefore, identifying novel markers and genes that regulate only the later stages of osteoclastogenesis while maintaining the ability of osteoclasts to differentiate may keep osteoblast and osteocyte signaling intact within the bone microenvironment.
